# Risk factors for dementia after critical illness in elderly medicare beneficiaries

**DOI:** 10.1186/cc11901

**Published:** 2012-12-17

**Authors:** Carmen Guerra, Walter T Linde-Zwirble, Hannah Wunsch

**Affiliations:** 1 Department of Anesthesiology, College of Physicians & Surgeons, Columbia University, 622 West 168th Street, New York, NY, 10032 USA; 2 ZD Associates, 904 Deerfield Place, Perkasie, PA, 18944 USA; 3 Department of Epidemiology, Mailman School of Public Health, Columbia University, 722 West 168th Street, New York, NY, 10032 USA

## Abstract

**Introduction:**

Hospitalization increases the risk of a subsequent diagnosis of dementia. We aimed to identify diagnoses or events during a hospitalization requiring critical care that are associated with a subsequent dementia diagnosis in the elderly.

**Methods:**

A cohort study of a random 5% sample of Medicare beneficiaries who received intensive care in 2005 and survived to hospital discharge, with three years of follow-up (through 2008) was conducted using Medicare claims files. We defined dementia using the International Classification of Diseases, 9th edition, clinical modification (ICD-9-CM) codes and excluded patients with any prior diagnosis of dementia or cognitive impairment in the year prior to admission. We used an extended Cox model to examine the association between diagnoses and events associated with the critical illness and a subsequent diagnosis of dementia, adjusting for known risk factors for dementia.

**Results:**

Over the three years of follow-up, dementia was newly diagnosed in 4,519 (17.8%) of 25,368 patients who received intensive care and survived to hospital discharge. After accounting for known risk factors, having an infection (adjusted hazard ratio (AHR) = 1.25; 95% CI, 1.17 to 1.35), or a diagnosis of severe sepsis (AHR = 1.40; 95% CI, 1.28 to 1.53), acute neurologic dysfunction (AHR = 2.06; 95% CI, 1.72 to 2.46), and acute dialysis (AHR = 1.70; 95% CI, 1.30 to 2.23) were all independently associated with a subsequent diagnosis of dementia. No other measured ICU factors, such as need for mechanical ventilation, were independently associated.

**Conclusions:**

Among ICU events, infection or severe sepsis, neurologic dysfunction, and acute dialysis were independently associated with a subsequent diagnosis of dementia. Patient prognostication, as well as future research into post-ICU cognitive decline, should focus on these higher-risk subgroups.

## Introduction

A stay in an ICU is often associated with long-term sequelae that impact on rehabilitation and overall function [1-7]. Such sequelae involve physical and neuropsychological components and include post-traumatic stress disorder (PTSD), depression, and cognitive decline, including dementia [1-9]. For many patients, in particular the elderly, a hospitalization may be a turning point with regard to cognition, with a down-ward trajectory in the years following [10]. A few single site studies have examined general medical or surgical ICU patients [3,4,9] and demonstrated continued cognitive impairment several months after critical illness. A focus on cognitive decline in specific illness categories (for example, severe sepsis [8] or the Acute Respiratory Distress Syndrome (ARDS) [1,2,5,11] provides valuable incidence data in such patient groups; nonetheless, such studies lack the ability to determine independent risk factors in the broader ICU population.

Fear of developing dementia is strong in many older people, with uncertainty about the causes, anxiety regarding loss of self-identity and need for long-term care [12]. Although many risk factors for dementia in the general population are well characterized, such as increasing age and co-morbidities (for example, alcoholism, stroke, and Parkinson's disease [13]), we still know very little about events associated with critical illness that may increase the risk.

We hypothesized that specific diagnoses and ICU events could be identified as risk factors for a subsequent diagnosis of dementia. In particular, due to previous work demonstrating higher rates of cognitive decline in older people who develop severe sepsis [8], we hypothesized that infection in critically ill patients would be independently associated with a higher risk of dementia. In order to test these hypotheses we used a nationally representative sample of Medicare beneficiaries who survived critical illness to quantify the risk of a subsequent diagnosis of dementia and to examine specific characteristics of the critical illness that might be associated with development of dementia.

## Materials and methods

### Data source

This was a retrospective study using the Medicare Standard Analytic Files (SAF) from the Centers for Medicare and Medicaid Services (CMS). This dataset contains all fee-for-service claims, including hospital inpatient, hospital outpatient, skilled nursing facility, and 'carrier' claims (physician supplier part B files which includes all office visits), home health agency, and durable medical equipment for a random, longitudinal 5% sample of beneficiaries. We linked data from years 2004 through 2008 and derived the inception cohort from the 2005 sample.

### Cohort

**Figure 1:**
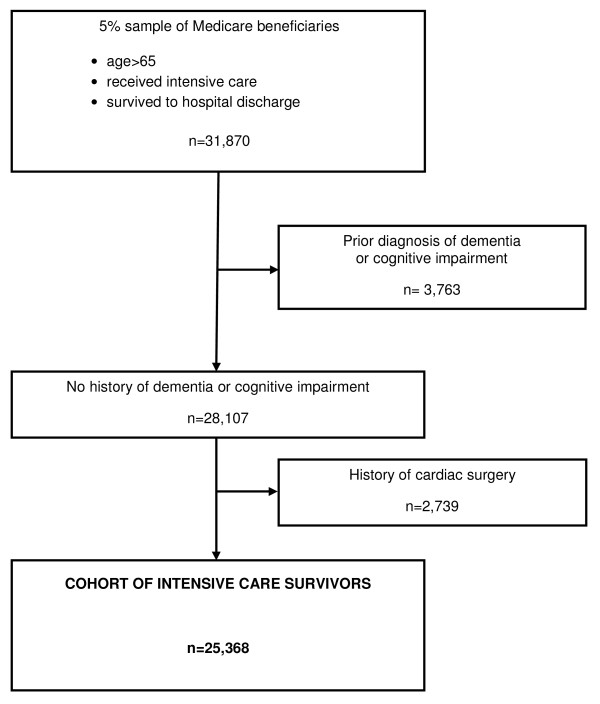
**Flowchart of exclusions and final study cohort**.

Our cohort consisted of a random 5% sample of all Medicare beneficiaries, ≥ 66 years old, who received intensive care during their hospitalization and survived to hospital discharge. We excluded all patients discharged to hospice care and patients who died in the same quarter (three months) of their discharge date. Because cardiac surgery may distinctly alter a patient's risk for dementia [14,15], we omitted anyone who had any history of cardiac surgery during the index hospitalization or in the year prior to admission (International Classification of Diseases, 9th revision, clinical modification (ICD-9-CM) codes 35.x, 36.x, and 39.61). We also excluded patients who only required intermediate intensive care. We used ICD-9-CM codes from data for all health care encounters in the year prior and during the index quarter to identify and exclude patients with any diagnosis for dementia (290.x, 294.x, 331.x,797.x) [14,16] or with any diagnosis for mild cognitive impairment (331.83) or general symptom mental loss (780.93) (see Figure 1) [17].

### Dementia assessment following discharge

An ICU survivor was identified as having dementia when there was a diagnosis for dementia (ICD-9-CM codes: 290.0 to 290.4, 294.0, 294.1, 294.8, 331.0, 331.1, 331.2, 331.7, 797.X) [16,17] recorded from fee-for-service claims in the subsequent three years of follow-up. We analyzed patient data for the three years following hospital discharge (2006 to 2008). Prior studies showed that three years of follow-up claims data is sufficient to identify dementia in a Medicare sample [16]. For privacy requirements, exact dates of hospital discharge and doctor visits were not available in the SAF - only the quarter of the year. We calculated time to diagnosis of dementia and death as the number of quarters (three month intervals) after the discharge quarter. The quarter in which the first diagnosis of dementia occurred was used as the incident quarter to mark the diagnostic onset for that individual.

### Patient characteristics and critical illness

The primary objective of this analysis was to evaluate specific aspects of the index critical illness. We examined the frequency of mechanical ventilation (ICD-9-CM codes 96.7x or tracheostomy 31.1 or Diagnosis Related Group 483) [18], and the type of patient (medical or surgical based on Diagnosis Related Groups). We also assessed acute organ dysfunctions (cardiac, respiratory, renal, neurologic, and hepatic) and diagnoses of infection and severe sepsis, using a standard definition [19]. Neurologic dysfunction consisted of anoxic brain damage, encephalopathy, and transient mental disorders. We also examined hemodialysis (39.95); in order to distinguish renal replacement therapy (RRT) for patients with acute renal failure we excluded patients who had RRT (based on fee-for-service claims ICD-9-CM code 39.95, and CPT code 90999) or a diagnosis of chronic renal failure (ICD-9 code 585.x, 586.x and CPT code G0327, G0323, G0317 to G0319) in the previous quarter. We calculated other characteristics of the index hospitalization including the ICU length of stay (based on the number of days billed for intensive care) and the hospital length of stay (days). Using data from the year prior, as well as the quarter of the index hospitalization, we identified potential confounders as specific diseases and conditions known or suspected to be related to dementia [see Additional file 1] [20].

### Data analyses

We calculated summary statistics for demographic and clinical characteristics using percentages, means (± standard deviation (SD)) and medians (with interquartile range (IQR)) as appropriate for the entire sample. We calculated the cumulative incidence of a diagnosis for dementia during follow-up using a cumulative incidence competing risks (CICR) method [21], since mortality precludes subsequent diagnosis of dementia and using the Kaplan-Meier method for a time-to-event analysis would overestimate the rate of dementia during the follow-up [22]. Follow-up for each participant started with the quarter after discharge and continued until the first diagnoses of dementia, death, or end-of follow-up (three years). Using the Kaplan-Meier method, we also quantified the overall three year mortality for the cohort.

We examined the associations between demographic variables, related diseases/conditions, and measures of critical care with subsequent diagnosis of dementia in ICU survivors using an extended Cox model with mortality censored and time-dependent covariates. We first used univariate analysis to examine the association between individual covariates and a diagnosis of dementia. We categorized age into five-year intervals, grouped race as non-Hispanic White, non-Hispanic black, and 'others' (Hispanic and other), and examined ICU and hospital length of stay using quartiles. We assessed acute organ dysfunctions both individually and as a count of 0, 1, and 2+ organ dysfunctions and assessed mechanical ventilation both as 'any' versus 'none', and categorized as less than 96 hours (96.71) and 96 hours or more (96.72) versus 'none'. We also created a composite variable with exclusive categories for any infection (no severe sepsis - defined as infection but no acute organ failure) and severe sepsis (infection with acute organ failure). We used a stepwise selection to create a parsimonious multivariable model for diagnosis of dementia for all ICU survivors by first adjusting for demographic characteristics and known risk factors for dementia and then individually evaluated the factors related to the episode of critical care. Covariates that were statistically significant (*P *< 0.05) were kept in the model. We examined possible collinearity among our critical illness markers by checking for a change in effect size. We assessed the Cox proportionality assumption graphically and added time-dependent covariates where the effect of a covariate varied during follow-up.

After identification of specific variables independently associated with a subsequent diagnosis of dementia, we used the Kaplan-Meier method to quantify the magnitude of the (unadjusted) differences in rates of diagnosis of dementia, with censoring for death or loss to follow-up.

We performed sensitivity analyses to assess the robustness of our findings. We excluded (1) patients with any hospitalization in the year prior to the index hospitalization with critical illness to minimize the impact of any prior illness and (2) patients with a known history of Parkinson's disease, head trauma, and alcohol abuse as these are strongly linked with subsequent development of dementia. We also assessed two sub-groups who represented patients with a greater overall severity of illness: (1) survivors who had at least three days of intensive care; and (2) survivors who received mechanical ventilation. Database management and statistical analyses were performed using Excel (Microsoft, Redmond, WA, USA), and SAS version 9.2 (SAS Institute Inc., Cary, NC, USA) software. This research was reviewed by the Columbia University Medical Center Institutional Review Board (IRB AAAE9908); due to the use of de-identified data it was deemed not human subjects research under 45 CFR 46.

## Results

### Baseline characteristics

**Figure 2:**
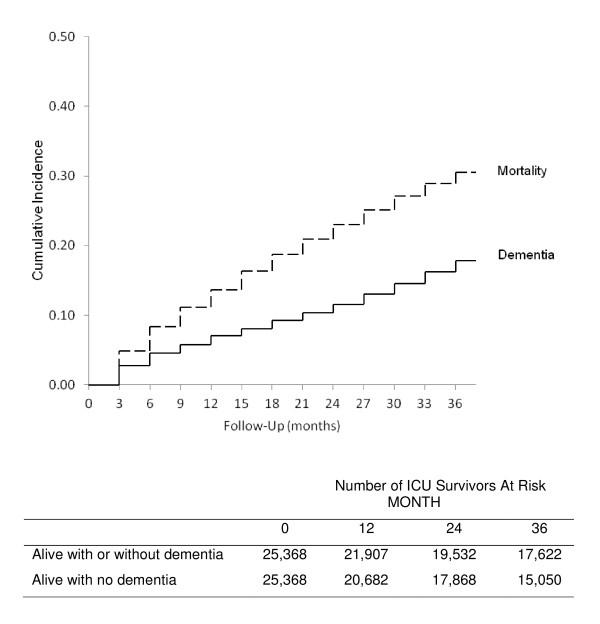
**Cumulative incidence of all mortality and dementia for elderly ICU survivors over three years, adjusting for mortality as a competing risk**. The dashed line is the cumulative incidence of all mortality during follow-up. The solid line is the cumulative incidence of dementia after adjusting for mortality as a competing event.

After exclusions, the cohort included 25,368 ICU survivors (Figure 1). The average age was 76.6 ± 6.8 years (Table 1). In the cohort, 89.1% were non-Hispanic white, and 52.3% were female. Of all ICU survivors 18.1% had at least one other hospitalization in the prior year and 2.5% had at least one other ICU stay in the prior year. The median ICU length of stay was two days with an IQR one to five days; the median overall hospital length of stay was six days, IQR three to ten days. The average duration of follow-up was 2.5 years, and the overall three year mortality for the cohort was 30.5%, with almost half of all deaths (45%) occurring in the first year (Table 1 and Figure 2). Dementia was diagnosed in 4,519 (17.8%) of all ICU survivors by the end of the three years of follow-up.

**Table 1:** Characteristics of critically ill patients who survived to hospital discharge.

	ICU Cohort (*n *= 25,368)
	Number (%)
**Index Hospitalization**	
Age, years: mean ± SD	76.6 ± 6.8
Female	13,275 (52.3)
Race	
non-Hispanic, white	22,602 (89.1)
non-Hispanic, black	1,830 (7.2)
Hispanic	407 (1.6)
Other	529 (2.1)
ICU length of stay, days: median (IQR)	2 (1 to 5)
Hospital length of stay, days: median (IQR)	6 (3 to 10)
Mechanically Ventilated	
None	23,067 (90.9)
Mechanically Ventilated	2,301 (9.1)
**Past Hospitalization History (prior year)**	
Any previous ICU admission	624 (2.5)
Any previous hospitalization	4,590 (18.1)
Number of previous hospitalizations	
0	20,778 (81.9)
1	3,035 (12.0)
2+	1,555 (6.1)
**Prior Diagnoses (prior year)^a^**	
None	8,450 (33.3)
Any potential risk factors for dementia	16,918 (66.7)
**Follow-Up**	
Total follow-up time, years	63543
Average follow-up time, years: mean ± SD	2.5 ± 0.9
Diagnosis of Dementia	4,519 (17.8)
Mortality	
1 year	3,461 (13.6)
2 year	5,836 (23.0)
3 year	7,746 (30.5)

^a^Based on ICD-9-CM codes. Full list of diagnoses are available in the Additional file 1. ICD-9-CM, International Classification of Diseases, 9th revision, clinical modification.

### Variables associated with a diagnosis of dementia

**Figure 3:**
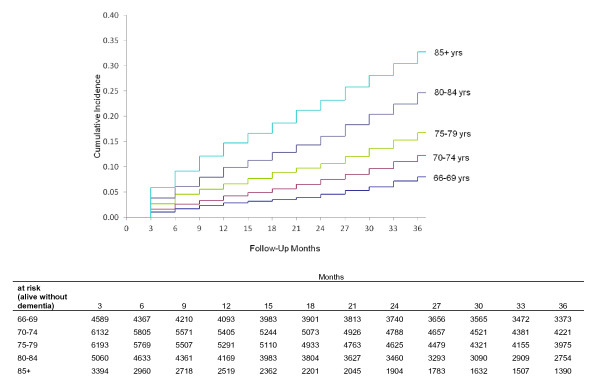
**Cumulative incidence of dementia by five year age categories**. Cumulative incidence of dementia, adjusted for mortality as a competing event, by age.

The age of patients was strongly associated with receiving a subsequent diagnosis of dementia in the three years following hospital discharge (Figure 3). We examined the univariate associations between factors that were related to the hospitalization for critical illness and subsequent diagnoses of dementia (Table 2). Rates for diagnosis of dementia were higher among medical compared with surgical patients (Hazard Ratio (HR) 1.75; 95% CI, 1.65 to 1.85). Mechanical ventilation ≥ 96 hours was associated with an increased risk of a diagnosis of dementia (HR 1.25; 95% CI 1.05 to 1.49) as was the need for acute RRT (HR 1.61; 95% CI 1.26 to 2.05). There was a stepwise association with severity of infection: infection alone (HR 1.44; 95% CI 1.34 to 1.54) and severe sepsis (HR 1.63; 95% CI 1.50 to 1.77). Extended length of ICU stay and hospital length of stay were both associated with an increased risk for diagnosis of dementia (HR 1.44; 95% CI 1.33 to 1.57 for patients in the highest quartile of ICU length of stay versus the lowest; HR 1.70; 95% CI 1.56 to 1.84 for the highest quartile of hospital length of stay versus the lowest).

**Table 2:** Frequency of dementia diagnoses in three years of follow-up by characteristics from the index hospitalization.

	Total Cohort, number	Dementia in 3 years follow-up, number (%)	HR	(95% CI)	*P *Value
Total cohort	25,368	4,519 (17.8)			
Surgical	12,740	1,780 (14.0)	1 (ref)	-	-
Medical	12,628	2,739 (21.7)	1.75	(1.65, 1.85)	< .01
					
Mechanical Ventilation					
None	23,067	4,108 (17.8)	1 (ref)	-	-
< 96 hours	1,716	301 (17.5)	1.09	(0.97, 1.22)	0.17
≥ 96 hours	585	110 (18.8)	1.25	(1.05, 1.49)	0.01
					
No Acute RRT	25,094	4,453 (17.8)	-	-	-
Acute RRT	274	66 (24.1)	1.61	(1.26, 2.05)	< .01
					
No Infection	17,151	2,783 (16.2)	1 (ref)	-	-
Infection only^a^	5,072	1,053 (20.8)	1.44	(1.34, 1.54)	< .01
Severe sepsis	3,145	683 (21.7)	1.63	(1.50, 1.77)	< .01
					
Acute Organ Dysfunction					
None	19,165	3,289 (17.2)	1 (ref)	-	-
One	5,003	973 (19.5)	1.25	(1.16, 1.34)	< .01
Two or more	1,200	257 (21.4)	1.43	(1.26, 1.62)	< .01
Specific Organ Dysfunction^b^					
Cardiac	1,888	351 (18.6)	1.08	(0.97, 1.20)	0.18
Respiratory	2,164	394 (18.2)	1.17	(1.05, 1.30)	< .01
Renal	2,194	464 (21.2)	1.37	(1.24, 1.51)	< .01
Neurologic	351	130 (37.0)	2.38	(2.00, 2.84)	< .01
Encephalopathy	144	53 (36.8)	-	-	-
Delirium	167	68 (40.7)	-	-	-
Anoxia	48	12 (27.1)	-	-	-
Hematologic	940	176 (18.7)	1.12	(0.96, 1.30)	0.14
					
ICU length of stay (quartiles)^c^					
1	8,255	1,290 (15.6)	1 (ref)	-	-
2	5,349	977 (18.3)	1.23	(1.13, 1.34)	.04
3	6,748	1,270 (18.8)	1.31	(1.21, 1.41)	.11
4	4,953	971 (19.6)	1.44	(1.33, 1.57)	< .01
					
Hospital length of stay (quartiles)^d^					
1	7,201	1,068 (14.7)	1 (ref)	-	-
2	6,848	1,218 (17.8)	1.30	(1.20, 1.41)	< .01
3	5,640	1,085 (19.2)	1.47	(1.35, 1.60)	< .01
4	5,679	1,156 (20.4)	1.70	(1.56, 1.84)	< .01

^a^Bacterial or fungal infection but without acute organ failure (that is, no diagnosis of severe sepsis); ^b^hepatic failure, as a subtype of acute organ dysfunction, is not shown because of low frequency. The reference group for each of the five subtypes is everyone without that dysfunction; ^c^missing data on 63 patients; ^d^hospital length of stay includes ICU days. HR, hazard ratio; ref, reference; RRT, renal replacement therapy.

**Figure 4:**
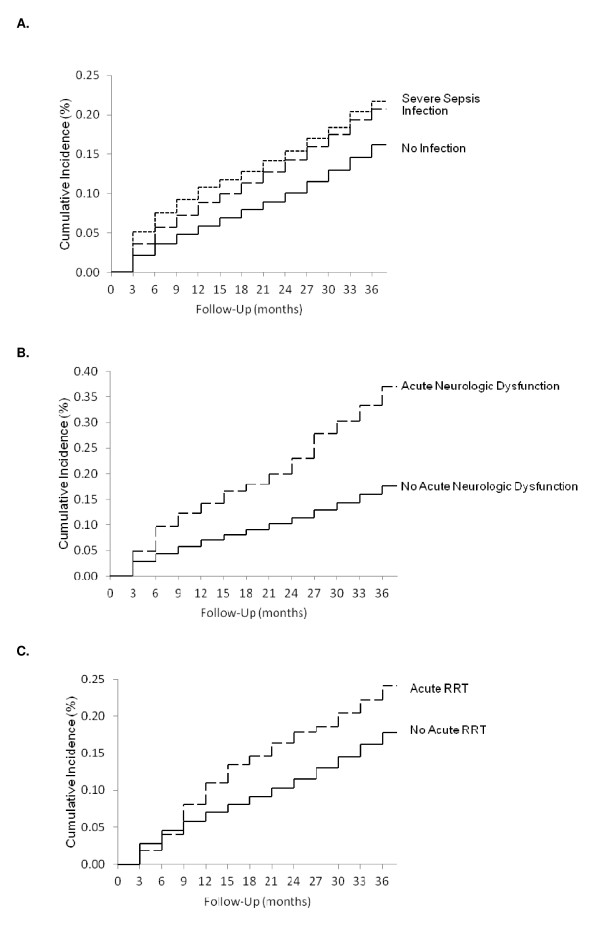
**Cumulative incidence of dementia, stratified by (A) infection or severe sepsis, (B) acute neurologic dysfunction, (C) acute renal replacement therapy**. Cumulative incidence (**A**. long dashed line is for infection, short dashed line is for severe sepsis, solid line is for no infection; **B**. dashed line is for neurologic dysfunction, solid line is for none; **C**. dashed line is for acute RRT, solid line is for none) of dementia after adjusting for mortality as a competing event. RRT, renal replacement therapy.

After multivariable adjustment, three factors associated with the hospitalization remained independently associated with a subsequent diagnosis of dementia (Table 3): (1) a critical illness with the presence of an infection (Adjusted Hazard Ratio (AHR) 1.25; 95% CI 1.17 to 1.35), with a higher risk associated with more severe infection (AHR for severe sepsis 1.40; 95% CI 1.28 to 1.53) (Figure 4A); (2) having acute neurologic dysfunction during critical illness (AHR 2.06; 95% CI 1.72 to 2.46) (Figure 4B); and (3) RRT for acute renal failure was a time-dependent risk factor that increased risk only after six months of follow-up (AHR 1.70; 95% CI 1.30 to 2.23) (Figure 4C). In addition, medical patients (AHR 1.41; 95% CI 1.33 to 1.50) continued to have an increase in risk as did patients with prior hospitalizations (AHR 1.36; 95% CI 1.27 to 1.46). No other factors associated with the critical illness, including mechanical ventilation or ICU length of stay, remained statistically significant; hospital length of stay was collinear with many of the critical care variables and was dropped from the analysis. We restricted our cohort to assess the robustness of our findings. All critical illness event markers remained significant and similar in magnitude among critical care survivors with no prior hospitalizations and for the sample that excluded patients with a history of Parkinson's disease, head trauma and alcohol abuse. We also assessed the survivors who had at least three days of critical care and found all markers significant but the magnitude of increased risk for a diagnosis of dementia was the same for infection and severe sepsis. Among survivors who were mechanically ventilated, only 2% had RRT for acute renal failure and RRT was no longer a significant marker for subsequent dementia, nor was having any prior hospitalization [see Additional file 2].

**Table 3:** Multivariate model of factors associated with dementia diagnosis during three years of follow-up.

	Multivariable Model		
	Adjusted HR	(95% CI)	*P *Value
**Index Hospitalization**			
No Infection	1 (ref)	-	-
Infection only^a^	1.25	(1.17, 1.35)	< .01
Severe sepsis	1.40	(1.28, 1.53)	< .01
			
None	1 (ref)	-	-
Acute neurologic dysfunction^b^	2.06	(1.72, 2.46)	< .01
			
No acute RRT	1 (ref)	-	-
Acute RRT (post 6 months)	1.70	(1.30, 2.23)	< .01
Acute RRT (6 months of follow-up)	0.73	(0.40, 1.33)	0.30
			
Surgical	1 (ref)	-	-
Medical	1.41	(1.33, 1.50)	< .01
			
None	1 (ref)	-	-
Any prior hospitalization	1.36	(1.27, 1.46)	< .01
			
Age 66 to 69 years	1 (ref)	-	-
Age 70 to 74 years	1.65	(1.45, 1.87)	< .01
Age 75 to 79 years	2.36	(2.09, 2.66)	< .01
Age 80 to 84 years	3.74	(3.33, 4.21)	< .01
Age 85+ years	5.37	(4.77, 6.05)	< .01
			
Non-hispanic, White	1 (ref)	-	-
Other races (hispanic and other)	1.18	(1.02, 1.36)	0.03
Non-hispanic, Black	1.78	(1.62, 1.97)	< .01
			
Male	1 (ref)	-	-
Female	1.17	(1.10, 1.24)	< .01
			
Cerebrovascular disease	1.53	(1.40, 1.68)	< .01
Depression	1.23	(1.08, 1.40)	< .01
Parkinson's Disease	2.31	(1.90, 2.80)	< .01
Alcohol Abuse	1.55	(1.18, 2.04)	< .01
Hypoglycemia (2 years of follow-up)	1.41	(1.12, 1.77)	< .01
Hypoglycemia (post 2 years of follow-up)	0.88	(0.57, 1.36)	0.56
Hypertension (6 months of follow-up)	1.08	(0.96, 1.22)	0.22
Hypertension (post 6 months of follow-up)	0.52	(0.48, 0.56)	< .01
Chronic renal failure (2 years of follow-up)	1.29	(1.11, 1.51)	< .01
Chronic renal failure (post 2 years of follow-up)	0.81	(0.62, 1.06)	0.12

^a^Bacterial or fungal infection but without acute organ failure (that is, no diagnosis of severe sepsis); ^b^acute neurologic dysfunction includes delirium, anoxia, and encephalopathy. Extended Cox-model adjusted for risk factors of dementia and time dependent coefficients, see online data supplement for full model, global null hypothesis likelihood ratio test *P *< .0001. HR, hazard ratio; ref, reference; RRT, renal replacement therapy.

## Discussion

Among elderly ICU survivors, a diagnosis of infection, particularly severe infection as defined by severe sepsis, acute neurologic dysfunction, and RRT for acute renal failure, were all independently associated with an increased risk of a subsequent diagnosis of dementia among a critically ill population, even after accounting for known risk factors for dementia. These findings suggest that certain diseases and events associated with a critical illness may increase the risk of dementia, or 'unmask' previously undiagnosed dementia [23]. The consequences of surviving a critical illness are broad, and may include increased risk of depression, PTSD, and physical debilitation that can be transient or permanent [1-5,7,8,11,24]. Use of a large administrative database of critically ill patients has allowed us to evaluate broadly all critically ill patients to determine which groups are at greatest risk for cognitive decline.

Hospitalization itself is clearly a risk factor for development of dementia [6]. Our findings are consistent with a recent study of cognitive impairment that focused only on patients with severe sepsis and found an increased risk of moderate/severe cognitive impairment following severe sepsis in a sample from the Health and Retirement Study [8], as well as data suggesting that post-operative delirium may be associated with longer term cognitive impairment [25]. Moreover, our findings extend the study by Ehlenbach *et al. *that demonstrated an increased rate of dementia among patients more than 65 years old who were admitted to the hospital for noncritical and critical illness [6], but had too small a sample size to identify specific risk factors in the critically ill population.

What has remained less clear is whether specific critical events during a hospitalization, such as mechanical ventilation, elevate the risk. We were able to determine that aspects of critical illness, such as the need for mechanical ventilation or the length of stay in an ICU, do not appear to add to the risk of dementia for elderly patients who experience a critical illness. The finding that there was no association with the need for mechanical ventilation is consistent with the work by Iwashyna *et al. *that found similar risks of cognitive impairment in severe sepsis patients with and without mechanical ventilation [8]. Our findings using a large cohort also replicate the conclusions from small cohorts of mechanically ventilated patients (with or without ARDS), which compared survivors of critical care with and without cognitive impairment and found no association between neurocognitive outcomes and length of mechanical ventilation, length of ICU stay, and length of hospital stay [2,3].

The mechanisms by which infection (particularly severe sepsis), acute neurologic dysfunction, and acute renal failure (defined by need for RRT) might increase the risk of dementia remain speculative and likely inter-related. One possibility, proposed by Jackson *et al. *is that those aspects of critical illness that incite a systemic inflammatory reaction, such as in sepsis or septic shock, may alter brain activity and start the development of cognitive impairment [26]. Recently, studies show dementia to be more common among people on RRT for chronic renal failure [27,28]; however, it is unclear whether the increased rate is attributable to the effects of hemodialysis or from direct effects of renal failure.

Data also suggest that many critically ill patients experience delirium at some point during the hospitalization [26]. Whether delirium is a harbinger of future dementia [29] or purely a marker for increased organ dysfunction and severity of illness remains unclear. Data on electro-encephalogram findings in critically ill patients demonstrate a high rate of sub-clinical seizure activity [30], suggesting that damage to the brain may often occur during a critical illness, such as sepsis. Recent studies have demonstrated a relationship between delirium in the ICU and subsequent cognitive dysfunction [31-34]. Moreover, patients with longer duration of delirium also appear to have smaller brain volume at hospital discharge and at 3-months of follow-up, which appears to correlate with worse cognitive performance at 12 months [32,33]. Neurologic damage and cognitive impairment can also occur during acute renal failure [35-37]. Additional studies focused on these higher risk groups during and after critical illness with follow-up, are needed to understand and potentially prevent these long-term sequelae of critical illness.

We found a strong association between the age of critically ill patients and subsequent diagnoses of dementia; our oldest survivors (age 85+) had a cumulative incidence of dementia of 33% over three years. This relationship with age is consistent with many population studies [38]. The overall incidence of newly diagnosed dementia after critical illness is higher in our cohort than reported population-based incidence rates among the elderly in the United States, as expected for a cohort at an overall increased risk due to hospitalization; the incidence per 1,000 person-years was two to three times higher than similar age-stratified cohorts [39-41]. However, accurately assessing diagnoses of dementia as well as risk factors remains challenging using administrative data and there are a number of limitations to this analysis. Because we relied solely on Medicare claims to identify dementia, we used a definition found to capture 82% of dementia cases identified with a neuropsychology battery [42] and also used three years of claims data; in another study it was found to have a sensitivity of 85% when compared to in-home dementia assessment by clinical staff [16]. Any missed diagnoses of dementia would likely bias our findings towards the null. It is important to acknowledge that the severity of dementia is unknown from Medicare claims and early dementia was found to be under-reported in claims data [42,43]. Thus, our findings mostly relate to moderate to severe dementia diagnoses after critical illness. We also performed a number of sensitivity analyses to determine whether our findings were consistent across sub-groups of more severely ill patients, with similar results in all of the models.

Prior work in a study with two years follow-up showed that cognitive functional impairment at baseline is a significant predictor of developing dementia [44]. While we excluded everyone with any diagnosis of cognitive impairment from our sample and adjusted for risk factors associated with dementia, we were limited in our ability to assess or control for cognitive status at baseline. It is possible that the events during the critical illness may reveal dementia that was present but had not yet been diagnosed. This is particularly likely for infection and severe sepsis, as the majority of the increase in diagnoses appeared to occur in the first few months after the hospitalization [23]. The recent work on pre- and post-sepsis burden of geriatric conditions by Iwashyna *et al. *suggests that we should be cautious in our interpretation of post-ICU morbidity without robust data on the pre-hospitalization trajectory [23,45]. Moreover, the increase in healthcare encounters associated with a critical illness may bring a cognitive problem to the attention of a healthcare provider. Finally, while we used a nationally representative sample and were able to adjust for clinical characteristics, we were not able to assess fully the severity of critical illness or quantify other possible measures of exposure to critical care, such as the exact length of mechanical ventilation and medications given. Diagnoses of delirium and encephalopathy during the hospitalization were included in our definition of acute neurological dysfunction, and appeared to have a strong relationship with a future diagnosis of dementia but we could not accurately assess the frequency of delirium during the stay, as defined in clinical studies of critically ill patients [46] or the characteristics of the encephalopathy. Moreover, early or mid-life factors may impact development of disease that occurs later, and these exposures could not be quantified [47-50].

Despite these limitations, this study provides important information regarding risk factors for subsequent diagnoses of dementia and is consistent with a growing body of literature regarding cognitive dysfunction after hospitalizations and among severe sepsis patients in particular. As the US population ages, millions of elderly people now survive a critical illness every year [51]. A greater understanding of the consequences of these hospitalizations and identification of patients at risk for specific types of morbidity as well as mortality may allow for better planning. Moreover, identification of groups at risk allows for studies targeted to high risk populations to identify potentially modifiable risk-factors for diseases such as dementia.

## Conclusions

Among ICU events, infection or severe sepsis, neurologic dysfunction and acute renal replacement therapy were independently associated with a subsequent diagnosis of dementia. Patient prognostication, as well as future research into post-ICU cognitive decline, should take account of these higher-risk subgroups.

## Key messages

• Prior studies suggest an increased risk of cognitive dysfunction or dementia associated with critical illness and/or sepsis

• This study used US Medicare data to determine which factors related to critical illness were associated with the increased risk of a subsequent diagnosis of dementia

• Among critically ill patients who survived to hospital discharge, infection or severe sepsis, neurologic dysfunction and the requirement for acute renal replacement therapy were all independently associated with an increased risk of a diagnosis of dementia in the following three years

• The use of mechanical ventilation and length of stay in the ICU were not independently associated with an increased risk of a diagnosis of dementia

## Abbreviations

AHR: adjusted hazard ratio; ARDS: acute respiratory distress syndrome; CICR: cumulative incidence competing risks; CMS: Center for Medicare and Medicaid Services; ICD-9-CM: International Classification of Diseases, 9th revision, clinical modification; PTSD: post-traumatic stress disorder; RRT: renal replacement therapy; SAF: Standard Analytic Files.

## Competing interests

The authors declare that they have no competing interests.

## Authors' contributions

HW acquired the data, participated in study concept and design, analysis and interpretation of data, statistical analysis, performed critical revision of the manuscript for important intellectual content and obtained funding. CG participated in study concept and design, analysis and interpretation of data, statistical analysis, drafted the manuscript and performed critical revision of the manuscript for important intellectual content. WTL-Z participated in study concept and design, analysis and interpretation of data, statistical analysis, provided technical support, and performed critical revision of the manuscript. The work was performed at Columbia University. All authors read and approved the final manuscript.

## Supplementary Material

Additional file 1 **ICD9 codes for conditions related to dementia**. A table of the ICD 9 codes we used to define diseases and conditions known or suspected to be related to dementia. Click here for file

Additional file 2 **Multivariable models for Sensitivity Analyses**. Multivariable models for four subgroups of our sample: (1) sample excluding patients with prior hospitalizations; (2) sample excluding patients with known Parkinson's disease, head trauma or alcohol abuse; (3) sample of mechanically ventilated patients only; and (4) sample of patients who received 3+ days of intensive care. Click here for file
