# Supplementary material for: Risk factors for dementia after critical illness in elderly medicare beneficiaries
Source: Crit Care. 2012 Dec 17;16(6):R233. doi: 10.1186/cc11901 (PMC3672622; doi:10.1186/cc11901)
Supplement: Additional file 1 — ICD9 codes for conditions related to dementia. A table of the ICD 9 codes we used to define diseases and conditions known or suspected to be related to dementia. [file cc11901-S1.PDF]

ICD-9-CM codes and sample sizes for diseases and conditions related to dementia

Disease/Condition	ICD-9-CM codes	ICU Cohort (n=25,368) No. (%)
**Hypertension**	401.0, 401.1, 401.9, 642.00-642.04, 437.2	12029 (47.4)
**Chronic Pulmonary disease**	490-492.8, 493.00-493.92, 494.0-494.1, 495.0-505, 506.4	5511 (21.7)
**Diabetes**	250.00-250.33, 250.40-250.93, 648.00-648.04, 775.1	5155 (20.3)
**Hypothyroidism**	243-244.2, 244.8, 244.9	2296 (9.1)
**Chronic Renal Failure**	585.3, 585.4, 585.5, 585.6, 585.9, 586, V420, V451, V56.0-V56.32, V56.8	1627 (6.4)
**Rheumatoid arthritis/collagen vascular diseases**	701.0, 710.0-710.9, 714.0-714.9, 720.0-720.9, 725	637 (2.5)
**Obesity**	278.0, 278.00, 278.01, 649.10-649.14, V85.30-V85.39, V85.4, V85.54, 793.91	818 (3.2)
**Weight loss**	260-263.9, 783.21-783.22	1320 (5.2)
**Fluid and electrolyte disorders**	276.0-276.9	4920 (19.4)
**Deficiency anemias**	280.1-281.9, 285.21-285.29,285.9	4598 (18.1)
**Alcohol abuse**	291.0-291.3, 291.5,291.8, 291.81, 291.82, 291.89, 291.9, 303.00-303.93, 305.00-305.03	322 (1.3)
**Drug abuse**	292.0, 292.82-292.89, 292.9, 304.00-304.93, 305.20-305.93, 648.30-648.34	67 (0.3)
**Depression**	300.4, 301.12, 309.0, 309.1, 311	1374 (5.4)
**Cerebrovascular accident**	430 - 434.9	1568 (6.2)
**Cerebrovascular disease**	435.0 - 438.9	2697 (10.6)
**Hypoglycemia**	251.0, 251.1, 251.2, 249.8, 250.8	593 (2.3)
**Head Trauma**	850.0 - 854.1, 800.1-800.4, 800.6-800.9, 801.1-801.4, 801.6-801.9, 803.1-803.4, 803.6-803.9, 804.1-804.4, 804.6-804.9	493 (1.9)
**Congestive heart failure**	398.91, 428.0-428.9	5261 (20.7)
**Valvular disease**	093.20-093.24, 394.0-397.9, 424.0-424.99, 746.3-746.6, V42.2, V43.3	2997 (11.8)
**Pulmonary circulation disorder**	415.11-415.19, 416.0-416.9, 417.9	1010 (4.0)
**Peripheral vascular disorder**	440.0-440.9, 441.00-441.9, 442.0-442.9, 443.1-443.9, 444.21-444.22, 447.1, 449, 557.1, 557.9, V43.4	3120 (12.3)
**Myocardial Infarction**	410.00-414.9	2772 (10.9)
**Epilepsy**	345.00-345.11, 345.2-345.3, 345.40-345.91	147 (0.6)
**Parkinson’s disease**	332.0	291 (1.2)
