# Supplementary material for: Risk factors for dementia after critical illness in elderly medicare beneficiaries
Source: Crit Care. 2012 Dec 17;16(6):R233. doi: 10.1186/cc11901 (PMC3672622; doi:10.1186/cc11901)
Supplement: Additional file 2 — Multivariable models for Sensitivity Analyses. Multivariable models for four subgroups of our sample: (1) sample excluding patients with prior hospitalizations; (2) sample excluding patients with known Parkinson's disease, head trauma or alcohol abuse; (3) sample of mechanically ventilated patients only; and (4) sample of patients who received 3+ days of intensive care. [file cc11901-S2.PDF]

Multivariable Models for Sensitivity Analyses

	Sample with No prior hospitalization		Sample with no Parkinson's disease, head trauma or alcohol abuse		Sample with 3+ days of intensive care		Sample mechanically ventilated	
	N=20,778		N=24,300		N=11,701		N=2,301	
	Adj HR (95% CI)	*P* Value	Adj HR (95% CI)	*P* Value	Adj HR (95% CI)	*P* Value	Adj HR (95% CI)	*P* Value
**Index Hospitalization**								
No Infection	1 (*ref*)		1 (*ref*)		1 (*ref*)		1 (*ref*)	
Infection only[a]	1.24 (1.14, 1.35)	<.01	1.26 (1.17, 1.36)	<.01	1.23 (1.11, 1.36)	<.01	--	
Severe sepsis	1.37 (1.24, 1.52)	<.01	1.40 (1.28, 1.53)	<.01	1.23 (1.11, 1.38)	<.01	1.31 (1.06, 1.62)	0.01
None	1 (*ref*)		1 (*ref*)		1 (*ref*)		1 (*ref*)	
Acute Neurologic dysfunction[b]	2.18 (1.79, 2.66)	<.01	2.19 (1.83, 2.63)	<.01	1.95 (1.55, 2.45)	<.01	2.49 (1.68, 3.71)	<.01
No acute RRT	1 (*ref*)		1 (*ref*)		1 (*ref*)		1 (*ref*)	
Acute RRT (post 6 months)	1.66 (1.22, 2.26)	<.01	1.68 (1.28, 2.21)	<.01	1.50 (1.03, 2.18)	0.03	0.64 (0.16, 2.63)	0.54
Acute RRT (6 month follow-up)	0.75 (0.39, 1.46)	0.40	0.72 (0.39, 1.35)	0.30	0.83 (0.41, 1.67)	0.60	0.75 (0.28, 2.05)	0.58
Surgical	1 (*ref*)		1 (*ref*)		1 (*ref*)		1 (*ref*)	
Medical	1.38 (1.29, 1.47)	<.01	1.41 (1.32, 1.50)	<.01	1.45 (1.33, 1.58)	<.01	1.27 (1.03, 1.54)	0.02
None	--		1 (*ref*)		1 (*ref*)		1 (*ref*)	
Any prior hospitalization	--		1.38 (1.28, 1.50)	<.01	1.20 (1.08, 1.33)	<.01	1.18 (0.93, 1.50)	0.17

Definitions of abbreviations: Adj HR = Adjusted Hazard Ratio; CI = confidence intervals; ref = reference; RRT=renal replacement therapy.

All models were adjusted for age, race, gender, cerebrovascular disease, depression, Parkinson disease, alcohol abuse, hypertension, hypoglycemia, and chronic renal failure. For the 2nd sensitivity model we did not include Parkinson disease or alcohol abuse as the sample excluded any people with history of those conditions. The sample mechanically ventilated had no one in the 'infection only' category.

[a] A bacterial or fungal infection but without acute organ failure (i.e. no diagnosis of severe sepsis).

[b] Acute neurologic dysfunction includes delirium, anoxic, and encepholopathy.
